# Droplet bubbling evaporatively cools a blowfly

**DOI:** 10.1038/s41598-018-23670-2

**Published:** 2018-04-19

**Authors:** Guilherme Gomes, Roland Köberle, Claudio J. Von Zuben, Denis V. Andrade

**Affiliations:** 1 Department of Physics and Interdisciplinary Science, São Carlos Institute of Physics (IFSC), University of São Paulo (USP), 13566-590 São Carlos-SP, Brazil; 2 Departament of Zoology, Institute of Biosciences (IB), São Paulo State University (UNESP), 13506-900 Rio Claro, SP Brazil

## Abstract

Terrestrial animals often use evaporative cooling to lower body temperature. Evaporation can occur from humid body surfaces or from fluids interfaced to the environment through a number of different mechanisms, such as sweating or panting. In Diptera, some flies move tidally a droplet of fluid out and then back in the buccopharyngeal cavity for a repeated number of cycles before eventually ingesting it. This is referred to as the bubbling behaviour. The droplet fluid consists of a mix of liquids from the ingested food, enzymes from the salivary glands, and antimicrobials, associated to the crop organ system, with evidence pointing to a role in liquid meal dehydration. Herein, we demonstrate that the bubbling behaviour also serves as an effective thermoregulatory mechanism to lower body temperature by means of evaporative cooling. In the blowfly, *Chrysomya megacephala*, infrared imaging revealed that as the droplet is extruded, evaporation lowers the fluid´s temperature, which, upon its re-ingestion, lowers the blowfly’s body temperature. This effect is most prominent at the cephalic region, less in the thorax, and then in the abdomen. Bubbling frequency increases with ambient temperature, while its cooling efficiency decreases at high air humidities. Heat transfer calculations show that droplet cooling depends on a special heat-exchange dynamic, which result in the exponential activation of the cooling effect.

## Introduction

Temperature exerts a profound influence on animal function^1^ and, therefore, most animals exhibit some degree of body temperature (*T*_b_) regulation, which involves balancing heat gain and loss with the environment, with variable contribution from metabolic heat production^2,3^. When *T*_b_ is elevated, either from environmental heat gain or from elevated metabolic heat production, animals typically promote heat dissipation to lower it^2^. Initially, heat dissipation can be fostered by changes in the organism´s thermal conductance, for example by changing body posture, altering the thickness of external insulation by erection or bristling of fur or feathers, and vasomotor adjustments^3–9^. In all cases, these responses maximize heat dissipation via dry radiative and conductive routes. However, if these responses are insufficient to maintain thermal homeostasis, terrestrial animals often resort to evaporative cooling to lower *T*_b_^2,4^. The high energy necessary for water vapourization removes heat from the animal´s body surface and, consequentially, promotes a highly effective way to lower *T*_b_. Evaporation can occur from humid body surfaces or animals can exhibit different modes of interfacing watery fluids to the environment. These include sweating in humans and a few other mammals, by triggering an increase in salivation rate and licking behaviour in some mammals^10^, or by promoting the evaporation from the upper airway mucosa through panting in most mammals and birds^10,11^. Among insects, however, evaporation from body surfaces is usually constrained by their chitinous exoskeleton and superficial wax covering^12,13^. Also, their tracheal respiratory system has a limited capacity to modulate heat dissipation^12,14,15^ and, since most insects are small, they are specially prone to gain heat rapidly in warm environments^12,15^. Finally, many insects are highly active and generate appreciable metabolic heat, which can contribute substantially to *T*_b_ elevation^14,15^. Accordingly, heat dissipation may pose important functional constraints to the regulation of *T*_b_ in insects. Herein, we demonstrate by infrared imaging of the oriental latrine blowfly, *Chrysomya megacephala*, that the behaviour known as bubbling in Diptera, in which flies moves tidally a droplet of crop fluid out and back in the buccal apparatus^16,17^, has an important and overlooked role in *T*_b_ regulation by promoting evaporative cooling^18–20^.

## Results and Discussion

**Figure 1:**
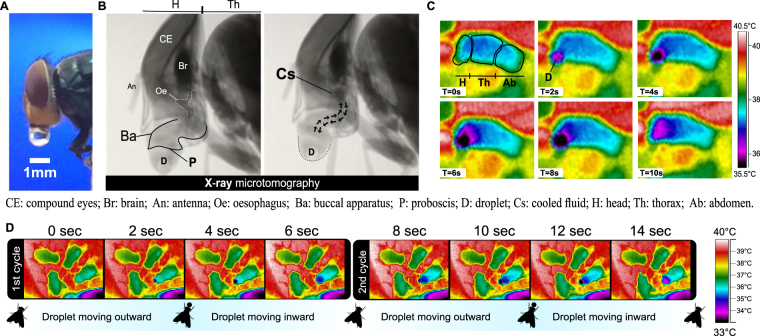
Imaging of the bubbling behaviour in the blowfly, *Chrysomya megacephala*. (**A**), Digital image of a fly holding a droplet of crop fluid with its mouthparts. (**B**), X-ray microtomography of a fly´s cephalic region showing mouthparts and the path of the fluid tidal movements (arrows). (**C**), Infrared side view imaging of a blowfly moving a droplet outward (top panel) and inward (lower panel) (T = time). (**D**), Infrared dorsal view of a group of 4 flies in which one of them (lower right) is moving a droplet. Movies showing the behaviour are provided in the Supplementary Information Videos 1–5.

**Figure 2:**
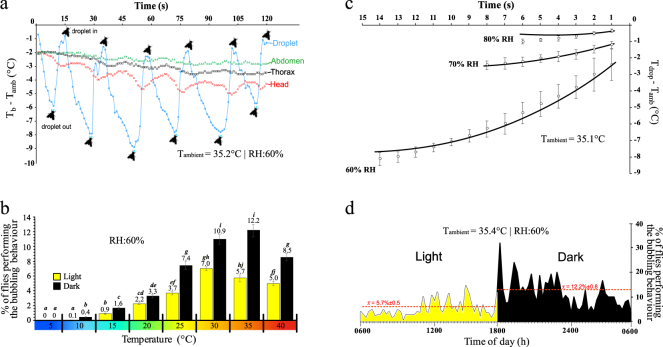
Temperature changes of different body parts of *C. megacephala* while displaying the bubbling behaviour and the influence of environmental parameters on the effectiveness and frequency of this behaviour. (**a**) Temporal changes in body and ambient temperature differentials for the crop fluid droplet and fly’s different body parts, along 6 cycles of droplet inward-outward movements taken from one individual fly. Evaporation readily cools the droplet upon its extrusion, while the lowering of the fly’s body progresses from head to abdomen, approximately in pace with the cooled droplet inward movement. (**b**) Percentage of flies performing bubbling behaviour under light (yellow) and dark (black) conditions at different temperatures. Bars and numbers depicts values averaged for 12 hours periods from 3 separate experimental trials. Significant differences among temperatures and between photophases are indicated by different letters (two-way ANOVA with Tukey’s test, *P* < 0.05 in all cases). (**c**) Effect of relative humidity on droplet temperature during the first outward movement of a bout of droplet tidal movement (*n* = 5). Empirical results obtained by infrared imaging (symbols and error bars) were in perfect agreement with those predicted by theoretical modelling (Lines, Extended Data Evaporative Cooling Model). Empirical results differed significantly among all different humidity levels (*P* < 0.0001, GLM). (**d**) Frequency of the bubbling behaviour over an entire circadian cycle averaged at 15 min periods, in 3 individual flies. Notice the marked increase in frequency at the transition between lighted and dark conditions, at 1800.

Blowflies accomplish evaporative cooling by tidally moving a droplet of fluid out and back into their buccopharyngeal cavity (Fig. 1 and Supplementary Information Videos 1 and 2). Infrared thermography revealed that an extruded droplet cools rapidly, depending on external conditions, down to as much as 8 °C below ambient temperature, within about 15 secs (Figs 1c, 1d and 2a). Blowflies then re-ingest the cooled droplet, which lowers the temperature of the fly´s head, thorax and abdomen by 1 °C, 0.5 °C, and 0.2 °C, respectively (Fig. 2a). As the cycle is repeated (see Fig. 2a), the temperature of the same body parts decreases by 3 °C, 1.6 °C, and 0.8 °C, respectively (Fig. 2a). The droplet fluid is composed by a complex mix of liquids from the ingested meal, enzymes from salivary glands, and antimicrobials associated to the foregut Diptera organ, the diverticulated crop^21^. This organ consists of lobes and ducts provided with pumping muscles and sphincters that, with the participation of mouthparts, drives the fluid’s inward-outward movements^21,22^. The cooled saliva moved inward can be pooled in the cephalic segment of the oesophagus juxtaposed to the brain (Fig. 1b, Supplementary Information Video 3), which is suggestive of the potential relevance of this behaviour for brain cooling. The droplet is moved in and out from 1 to 6 times before eventually being swallowed by the fly (Figs 1c, 1d and 2a). The frequency of evaporative cooling tends to increase with ambient temperature (Fig. 2b), as expected if bubbling was involved in the cooling process. This agrees with other instances in which evaporative cooling is recruited to promote heat dissipation^2^.

The use of the bubbling behaviour for evaporative cooling, however, does not relate uniquely to ambient temperature, but is also influenced by changes in activity level and heat production. At temperatures under 25 °C, *C. megacephala* is very active and flight activity requires the flight muscles to be warmed-up to be functional. During this time, evaporative cooling would cause an unwanted lowering in *T*_b_, and bubbling is seldom exhibited. At temperatures varying from 25 to 30 °C, the increased frequency of the behaviour reveals the increased importance of heat dissipation via evaporative cooling to *T*_b_ regulation. Finally, at temperatures above 30 °C, bubbling frequency tended to decrease (Fig. 2b - light), which is explained by the blowflies becoming inactive at these high temperatures. Particularly, as flight activity is almost suppressed at these temperatures, the reduction in flight muscles thermogenesis may alleviate the need to recruit the bubbling behaviour in order to keep the blowflies’ thermal homeostasis.

Still related to changes in activity level but unrelated to ambient temperature, we found that *C. megacephala* uses the bubbling behaviour to lower *T*_b_ while at night. Indeed, this diurnally active fly^23^ when exposed to dark conditions exhibit a significant increase in the frequency of this behaviour compared to what occurs at identical but lighted conditions (See Figs 2b, 2d and Supplementary Information Video 4). This response becomes particularly pronounced as temperature increases. For example, at 35 °C, the average number of flies displaying the behaviour increases from 5.7% under light, to 12.2% at dark (Figs 2b and 2d). The highest frequency occurs immediately after the transition from light to dark (Fig. 2d, Supplementary Information Video 4). The lowering of *T*_b_ during inactivity is likely to be advantageous to the flies as, in these animals, metabolic expenditure declines with temperature.

Evaporative cooling depends on relative humidity (RH) and temperature, as both of these variables affect the potential for water vaporization. When RH is increased from 60% to 70%, and then 80% (air temperature of 35 °C) the effectiveness of cooling markedly diminishes and, in fact, becomes almost ineffective at RH >70% (Fig. 2c). These results demonstrate that droplet cooling derives from the heat removal associated to liquid-vapour phase transition. Consistent with this, at very high relative humidities, flies do not even re-ingest the droplet, and, instead, spit it out (Supplementary Information Video 5).

**Figure 3:**
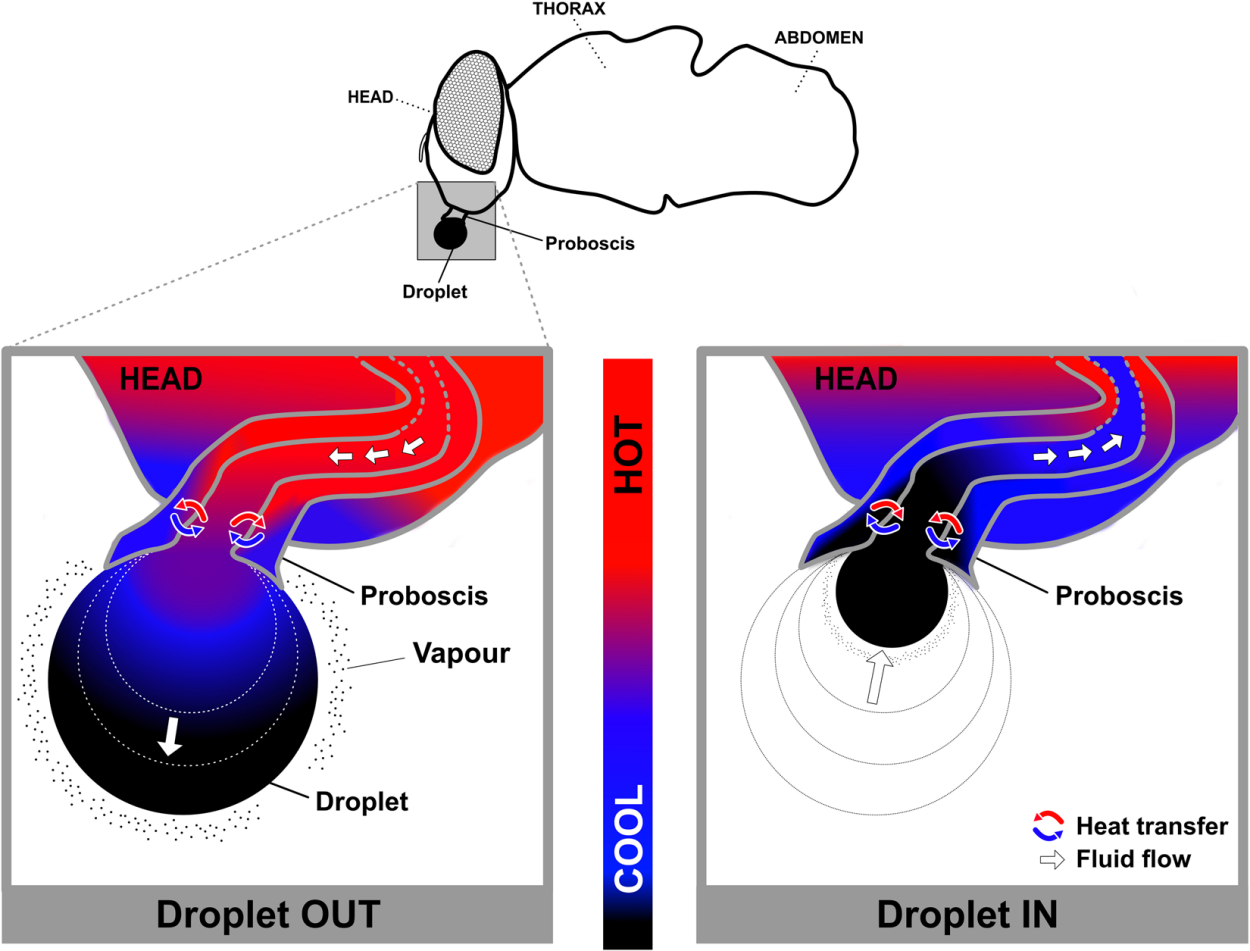
Schematic representation of the heat exchange dynamics as a crop fluid droplet is moved out and back in of the buccopharyngeal cavity of the blowfly, *Chrysomya megacephala*. This dynamic is proposed to result in an exponential activation that augments the effectiveness of evaporative cooling (for details see Extended Data: Heat transfer modeling).

**Figure 4:**
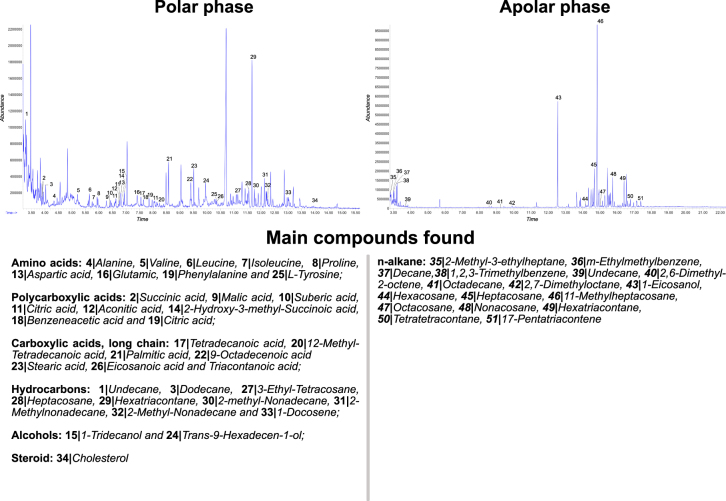
*Chrysomya megacephala* droplet fluid chemical composition. Gas chromatography mass spectrometry (GC-MS) analysis of polar and non-polar compounds present in *C. megacephala* crop fluid. GC-MS profile on top row and list of corresponding compounds.

The magnitude of the temperature change observed for the droplet fluid while being manipulated by the blowflies is greater than theoretical estimates for a similar but static droplet, under the same conditions. This observation indicates that the dynamics of droplet manipulation enhances evaporative cooling. By combining information on the droplet movement during its manipulation, obtained with x-ray microtomography, with the dynamics of temperature change, tracked with infrared thermography (see Fig. 1), we developed a theoretical model based on energy conservation (for details see Extended Data: Heat transfer modeling) that explains accurately the observed changes in the droplet temperature. The temperature changes predicted by our model are in perfect agreement with the empirical results obtained at different RH levels (see Fig. 2c). In this model, water vaporization cools droplet´s fluid immediately as it starts to expand on the outside part of the buccal apparatus. Due to the conduction within the proboscis, cooling spreads internally and lowers the temperature of the fluid that is about to be ejected into the forming droplet (Supplementary Information Videos 1 and 2). As this cooled fluid is injected into the droplet, the dynamics just described repeats in a positive feedback loop causing the potential activation of the process. As the cooled droplet fluid is moved inward, it lowers the temperature of the fly’s body tissues (Figs 1, 2a, 3 and Videos 1 and 2). Finally, we considered the possibility that volatile compounds could be added to the crop fluid, which could facilitate evaporative cooling, as such compounds have lower heat of vaporization than water^24^. Gas Chromatography Mass Spectrometry (GC-MS) revealed that the droplet fluid extruded by *C. megacephala* is composed by a diverse mixture of more than 50 chemical compounds, predominantly water, amino acids, hydrocarbons, polycarboxylic and carboxylic acids (Fig. 4). However, volatile compounds were a minor constituent (Fig. 4) of the droplet fluid. This observation, therefore, adds further support to our exponential activation model.

Many mammals lick saliva on their limbs – facilitating evaporative cooling – when heat stressed^2,10^. However, the thermoregulatory use of a droplet of fluid dynamically manipulated by the buccal apparatus to attain the benefits of evaporative cooling may be limited to small organisms, such as insects, since for larger animals mechanical constraints would impede the manipulation of a correspondingly sized fluid filled droplet. Indeed, behaviours sharing resemblance to bubbling have been associated to evaporative cooling in bumblebees^25^, hawk moths^26^, sawfly larvae^27^, and mosquitoes^28^. The thermoregulatory function of bubbling in Diptera, although suggested in the past^18^, was almost entirely discredited later on in favour of a more widely accepted role in food-processing^17,19,20,29^. Our study, however, provides strong evidence for the fact that bubbling does indeed play a relevant role in *T*_b_ regulation. Under given contexts, thermoregulatory considerations may become more relevant in driving this behaviour than food-processing, for example, we recorded that flies exhibit the bubbling behaviour at greater frequency when inactive at night, a period in which they are not feeding and food processing demand is probably lowered. In conclusion, we argue that the food-processing and the thermoregulatory role of the bubbling behaviour are not mutually exclusive and may overlap to different extent to accommodate compromises among feeding, activity, thermoregulation and other physiological and environmental determinants.

## Methods

### Animals

All experiments were performed on F2 generation of *Chrysomya megacephala* from a colony founded by flies captured in nature in southeastern Brazil. Animals were maintained in captivity under conditions described elsewhere^30^ and fed, at the larval phase, with an oligidic diet^31^, which provides a standardized developmental rate. In all experiments, we used only newly emerged adult flies (7 to 15 days post-metamorphosis), of undetermined sexes, and apparently healthy. For handling, we immobilized the flies by cooling them for 5 min at 4 °C.

### Experimental Protocol

We used IR thermography (detailed below) to monitor changes in surface body temperature in groups of 50 flies confined inside a circular PVC chamber (10 cm of diameter by 50 cm in length) attached, on one side to the thermal camera visor, and capped on the opposite side with a lycra screen. Usually, flies would remain on top of the lycra screen, which was convenient for focusing purpose. The animal container was then positioned inside a temperature-controlled chamber (Eletrolab - model EL011E) for a continuous period of 36 hours (12 hours for acclimation and 24 hours for data collection) under constant temperature and photoperiod of 12:12 H (Supplementary Figure 1). During this period, flies had free access to a sugary solution to feed, and we continuously monitored surface body temperature and quantified occurrences of the bubbling behaviour. To test for the influence of ambient temperature on the frequency and effectiveness of droplet extrusion, we monitored (as above) different groups of flies, at 5, 10, 15, 20, 25, 30, 35, and 40 °C, at relative humidity fixed at 60%. To test for the influence of ambient relative humidity, we fixed temperature at 35 °C and manipulated relative humidity level at 60, 70, and 80%. Each treatment was repeated at least three times. During the experiments, we recorded air temperature inside the chamber and inside each container [type K thermocouples connected to a data logger (RDXL4SD - Omega Engineering)]. Relative humidity inside the chamber was recorded (RHT10 - Extech).

### Infrared Thermography

Infrared images were acquired with a SC640 IR visor (Flir Systems) at 1 frame.sec^−1^, thermal sensitivity of 30 mK, and image resolution of 640 × 480 pixels. All temperature readings were automatically corrected assuming an emissivity of 0.98^32^, which was further confirmed by imaging black electric tape. Data collection and image processing were done using subroutines of the software ThermaCam 2.9 (Flir Systems).

### Gas Chromatography Mass Spectrometry (GC-MS)

Crop fluid droplets voluntarily exteriorized by *C. megacephala* were hand harvested (glass micro-capillary tube, ~1 mm diameter) from 100 live individual flies and stored at −70 °C until analysis. Polar and non-polar fractions were separated by the addition of cyclohexene (50 µl) to a crop fluid volume equaling 80 µl, followed by magnetic mixing and centrifugation. Immediately thereafter, the supernatant phase (~25 µl) was carefully aspirated with a micro-syringe under a stereo-microscope, thus yielding the non-polar fraction of the crop fluid. The polar fraction was sampled (20 µl) from the lower phase of the separation procedure just described. This fraction was then subjected to the ethyl chloroformate derivatization procedure^33–35^. Spectral mass determinations were read on a CG-MS analyzer (Agilent CG-6890; detector 5973 N; automatic injector 7683). The non-polar fraction of the crop fluid sample was analyzed using a VF-5ms column (Varian, CP9013, 30 m, 250 µm × 0.25 µm, 0.80 ml.min^−1^ He, 70 °C 0.1 min, 15 °C.min^−1^ up to 330 °C, injector 270 °C, volume 4 µl, splitless pulse, TIC 40 at 800 µma). The polar fraction of the crop fluid was analyzed using a VF-5ms column (Varian, CP9013, 30 m, 250 µm × 0.25 µm, 0.80 ml.min^−1^ He, 70 °C 0.1 min, 20 °C.min^−1^ to 330 °C, injector 275 °C, volume 3 µl, splitless pulse, TIC 40 a 800 µma. Data acquisition and spectral profiles analysis were performed by the MSD (ChemStation D.02.00.275 Agilent) and AMDIS (Automated Mass Spectral Deconvolution and Identification System) software and compared to the NIST database.

### Microtomography

Micro CT images of live and recently dead flies were attained to reveal the path of fluid movement. Flies were fixed with bee’s wax on a wood support, which was then inserted into the chamber of a microtomography scanner (Bruker, model SKYSCAN 1272). Immediately thereafter, image acquisition was initiated at 20-100 kV, 10 W, < 5 µm spot size at 4 W and detectability varying from 35 to 45 µm. All experiments were run at room temperature (~20 °C). Images were processed with the software CTVox (Bruker).

### Statistical analysis

Unless otherwise stated, all data are presented as mean ± S.E.M. Infrared images acquired during the 24 hours of recording were used to quantify the proportion of individual flies displaying the bubbling behaviour over 15 min time frames. Although the total number of flies being monitored were 50, at any given time a variable number of individuals would come in or out of the viewing field of the IR camera. Thus, the proportion of animals displaying the behaviour was calculated in relation to the total number of flies within the viewing field, at any given census. Once we quantified the serial 15 min frequencies over the 24 hours period, we used them to calculate a grand mean for each photophase (light vs dark) and for each experimental temperature (for the entire 24 hours period). Finally, since we had at least three replicates for each treatment, we averaged the data accordingly, after have confirmed that no significant difference existed among them (GLM test, 80%: F = 1.1; p = 0.3658; 70%: F = 2.2; p = 0.0888; and 60%: F = 0.53; p = 0.8066. for all pairwise comparisons). Frequency data were arcsine transformed and compared among treatments with a two-way ANOVA, with photophase and temperature as factors, followed whenever adequate by a *posthoc* Tukey test.

To quantify the influence of relative humidity on the effectiveness of the bubbling behaviour, we initially selected 10 instances in which the behaviour could be quantified in sufficient detail for each level of humidity. Then, we determined the dynamic change in the temperature differential (droplet temperature *versus* ambient temperature) over the duration of the first bout of droplet exteriorization (Fig. 2c) and tested for the occurrence of significant differences using a generalized linear model (GLM).

## Electronic supplementary material

Video 1

Video 2

Video 3

Video 4

Video 5

Supplementary information
