# Supplementary material for: Droplet bubbling evaporatively cools a blowfly
Source: Sci Rep. 2018 Apr 19;8:5464. doi: 10.1038/s41598-018-23670-2 (PMC5908842; doi:10.1038/s41598-018-23670-2)
Supplement: Supplementary file 6 — Supplementary information [file 41598_2018_23670_MOESM6_ESM.docx]

Supplementary information - Heat transfer modeling

**Droplet bubbling evaporatively cools a blowfly**

**Guilherme Gomes^1,*^, Roland Köberle^1^, Cláudio J. Von Zuben^2^, and Denis V. Andrade^2,*^**

^1^Departamento de Física e Ciência Interdisciplinar, Instituto de Física de São Carlos (IFSC), Universidade de São Paulo (USP), 13566-590, São Carlos-SP, Brasil.

^2^Departamento de Zoologia, Instituto de Biociências (IB), Universidade Estadual Paulista (UNESP), 13506-900, Rio Claro - SP, Brasil *guigomes@gmail.com and denis@rc.unesp.br

**Heat transfer modeling**

Evaporative cooling associated to the bubbling behaviour by the blowfly, *Chrysomya megacephala*, involves the active extrusion of a droplet of fluid, whose size increases with time $t\geq0$. Consider a spherical droplet of radius $a(t)$, sitting on the fly’s mouthparts/proboscis - the labellum - and in contact with the air at an ambient temperature $T_{\infty}=38.6^{\circ}C$. Let us discretize the evaporative cooling process in time-steps of size $\delta t$. Suppose that at time $t_{1}>0$, the droplet radius and temperature being $a_{1},T_{1}$ respectively, with $T_{1}<T_{L}$, where $T_{L}$ is the labellum/proboscis temperature. We imagine the cooling process to proceed in three stages:

the outside droplet surface is in contact with the ambient air and evaporates, extracting the necessary heat from the labellum at a rate

|  | $\dot{Q}_{k}=4\pi kR(t_{1})(T_{L}-T_{1}),$ | (1) |
| --- | --- | --- |

where $k$ is thermal conductivity of the labellum. We characterize the contact with the labellum by some length $R(t)\leq a(t)$.

This cools the labellum to a temperature $T_{L}^{'}$ between $T_{L}$ and $T_{1}$: $T_{L}^{'}=z_{1}T_{L}+\tilde{z}_{1}T_{1}$, where $z_{1}$ is a parameter $0<z_{1}<1$, controlling the efficiency of this process and $\tilde{z}_{1}=1-z_{1}$.

As mass $\delta m$ of the fluid is extruded from inside the head, it contacts the labellum (at temperature $T_{L}^{'}$ ) on its way out and is cooled to a temperature $T^{'}$. Supposing that this process has some efficiency $z_{2}$ with $0\leq z_{2}\leq1$, we use equ.(1) to get an equation for $T^{'}$

|  | $z_{2}\dot{Q}_{k}=4\pi kz_{2}R_{1}(T_{L}-T_{1})=c_{p}\dot{m}(T_{L}^{'}-T^{'})=c_{p}\dot{m}(z_{1}T_{L}+\tilde{z}_{1}{T-T^{'})}_{1}-T^{'}),$ | (2) |
| --- | --- | --- |

where $c_{p}$ is the specific heat of the fluid and $\dot{m}=\frac{\delta m}{\delta t}$ is the rate at which mass is added to the droplet.

As $\delta m$ is incorporated into the droplet, the labellum/proboscis temperature rises back to $T_{L}$, while the droplet temperature decreases. This is consistent with the very small variations of head temperature shown in Fig. 2A (main text).

In this way, the labellum/proboscis acts as a conductor for heat transfer between the internal fluid and the outside droplet. The injection of the $T_{1}$-dependent mass $\delta m$ into the droplet closes the feedback loop and leads to the exponential activation of the whole process.

We compute1-3 the droplet’s temperature dependence starting from equ.(2), to get

|  | $T^{'}=z_{1}T_{L}+\tilde{z}_{1}T_{1}-\frac{4\pi kz_{2}R_{1}}{c_{p}\dot{m}}(T_{L}-T_{1}).$ | (3) |
| --- | --- | --- |

As $\delta m$ is being incorporated into the droplet, its mass increases from $m_{1}$ to $m_{2}=m_{1}+\delta m$ and its temperature $T_{2}$ satisfies

$$m_{2}T_{2}=(m_{1}+\delta m)T_{2}=m_{1}T_{1}+\delta m(z_{1}T_{L}+\tilde{z}_{1}T_{1}-\frac{4\pi kz_{2}R_{1}}{c_{p}\dot{m}}(T_{L}-T_{1})),$$

where we used equ.(3). This yields

|  | $m_{2}T_{2}-m_{2}T_{1}=\delta m\left( -T_{1}+z_{1}T_{L}+\tilde{z}_{1}T_{1}-\frac{4\pi kz_{2}R_{1}}{c_{p}\dot{m}}(T_{L}-T_{1}) \right)$ | (4) |
| --- | --- | --- |

or

|  | $m_{2}\frac{T_{2}-T_{1}}{\delta m}=\left( z_{1}-\frac{4\pi kz_{2}R_{1}}{c_{p}\dot{m}} \right)(T_{L}-T_{1}).$ | (5) |
| --- | --- | --- |

As the droplet expands from $m_{1}$ to $m_{2}$, its temperature decreases from $T_{1}$ to $T_{2}$.

As this process goes on, the droplet temperature decreases as:

$$T_{0}>T_{1}>T_{2}>...>T_{n}.$$

Eventually the droplet becomes too large for $\delta m$ to be effective as a cooling agent and the fly stops extruding. The droplet has reached its lowest temperature $T_{n}$. The fly now re-ingest the droplet cooling the fly’s body.

The whole process is continuous and we therefore take the continuum limit $\delta t\to0$ to get a differential equation for $T(t)$:

|  | $m\frac{\mathrm{dT}}{\mathrm{dm}}=\left( z_{1}-\frac{4\pi kz_{2}R}{c_{p}\dot{m}} \right)(T_{L}-T)$ | (6) |
| --- | --- | --- |

or

$$\frac{\mathrm{dT}}{T_{L}-T}=\left( z_{1}-\frac{4\pi kz_{2}R}{c_{p}\dot{m}} \right)\frac{\mathrm{dm}}{m}.$$

Noticing that $\frac{\mathrm{dm}}{m}=\dot{m}\frac{\mathrm{dt}}{m}$ we get

|  | $\frac{\mathrm{dT}}{T-T_{L}}=\left( \frac{\alpha z_{2}R(t)-z_{1}\dot{m}(t)}{m(t)} \right)dt.$ | (7) |
| --- | --- | --- |

with $\alpha=\frac{4\pi k}{c_{p}}.$ We integrate this to get for $\hat{T}(t)\equiv T(t)-T_{L}$

| $\hat{T}\left( t \right)=\hat{T}\left( 0 \right)e^{\int_{0}^{t} \frac{\alpha z_{2}R\left( t^{'} \right)-z_{1}\dot{m}\left( t^{'} \right)}{m\left( t^{'} \right)}dt^{'}}$ (8) |
| --- |

Notice the competition between the two terms in the integrand: increasing the $R$-dependent term promotes cooling, whereas an increasing droplet-size due to the $\dot{m}$-dependent term inhibits it. The minimum temperature $\hat{T}(t)$ is reached, when the two terms are of equal size.

The active manipulation of the evaporation process results in an exponential amplification: as droplet cools, the evaporation process becomes more efficient.

To estimate the effect of relative humidity on the cooling process, notice that in equ.(2) only the fraction $z_{2}$ is used in cool $\delta m$, we neglect several processes, one being the evaporation flux

|  | $\dot{Q}_{\mathrm{vap}}=4\pi aLD\left( c_{0}(T)-c_{\infty} \right),$ | (9) |
| --- | --- | --- |

where $L,D,c_{0},c_{\infty}$ are the latent heat of the fluid droplet, the diffusivity of water vapour in air, the vapour concentration at the droplet surface and the vapour concentration far away from it, respectively. The humidity $H$ is defined as $c_{\infty}=Hc_{s}$, where $c_{s}$ is the saturated vapour density far away from the fly. Since

$$\dot{Q}_{k}=z_{2}\dot{Q}_{k}+(1-z_{2})\dot{Q}_{k},$$

we set

|  | $\dot{Q}_{\mathrm{vap}}=(1-z_{2})\dot{Q}_{k}\equiv\tilde{z}_{2}\dot{Q}_{k}.$ | (10) |
| --- | --- | --- |

Setting $c_{0}(T)=c_{\mathrm{sat}}(T)$ and approximating $c_{\mathrm{sat}}(T)\sim c_{s}$, $\dot{Q}_{\mathrm{vap}}$ becomes proportional to $1-H$:

|  | $\dot{Q}_{\mathrm{vap}}=4\pi LDac_{s}(1-H)$ | (11) |
| --- | --- | --- |

We incorporate this effect changing $\alpha$ to $\alpha_{H}=\tilde{z}_{2}(1-H)\alpha$ in equ.(8).

To test the model just presented, we applied it to predict the changes in temperature of a realistic fluid droplet, under realistic conditions, as if it was manipulated by a fly at $35^{\circ}C$ and relative humidities of 80, 70, and 60%. In doing so, we accepted that:

The relaxation-time for an evaporating droplet with radius $a$ to reach its equilibrium temperature after changing the surrounding temperature is

|  | $\tau_{\mathrm{relax}}=\frac{a^{2}\rho_{d}c_{p,d}}{3k_{\mathrm{air}}[1+\frac{\mathrm{LD}}{k_{\mathrm{air}}}[\frac{\mathrm{dc}}{\mathrm{dT}}]_{s}}.$ | (12) |
| --- | --- | --- |

If we use water properties for the density $\rho$, the specific heat at constant pressure $c_{p,d}$, the latent heat $L$, the diffusivity of water-vapour in air $D$, the thermal conductivity of air $k_{\mathrm{air}}$ and the slope of a linear fit for $10^{\circ}C \leq T\leq35^{\circ}C$ to the saturated water-vapour pressure, considering a droplet of $a\sim1mm$, we get

|  | $\frac{\mathrm{LD}}{k_{\mathrm{air}}}[\frac{\mathrm{dc}}{\mathrm{dT}}]_{s}\sim3, \tau_{\mathrm{relax}}\sim14 sec.$ | (13) |
| --- | --- | --- |

The internal relaxation time $\tau_{i}$ for a cylinder-shaped volume $\pi r^{2}h$ with thermal diffusivity $\kappa=\frac{k}{\rho c_{p,d}}$ is

$$\tau_{i}=\frac{2\pi rh}{\kappa}.$$

For $r\sim0.1mm$ and $h=0.5mm$, we have

|  | $\tau_{i}\sim0.35 sec\gg r^{2}/\kappa_{\mathrm{air}}\sim0.05 sec$ | (14) |
| --- | --- | --- |

Thus, we get the following time-scales

|  | $\tau_{\mathrm{relax}}(air)\sim14 sec\gg\tau_{i}\sim0.35 sec\gg a^{2}/\kappa_{\mathrm{air}}\sim0.05 sec$ | (15) |
| --- | --- | --- |

We may therefore neglect non-equilibrium processes of the droplet and since $\kappa_{\mathrm{labellum}}\gg\kappa_{\mathrm{air}}$, the heat-conduction process through the labellum is fast enough to be considered instantaneous.

To fit our data we use water parameters $k=0.2*10^{-3}[\frac{W}{mm\cdot deg}],c_{p}=4.2[\frac{J}{g\cdot deg}]$.We assume $a(t),R(t)$ to be linear functions of $t$, which is the simplest possible hypothesis, requiring thus $4$ droplet-parameters. We furthermore have two material parameters $z_{1},z_{2}$. We used these six parameters to fit $\mathrm{DT}$ of equ.(8) to our experimental data for $H=0.7$. This yields $z_{1}=0.12,z_{2}=0.35$ and $a(t)$ expands from $a(t=0)=0.14 mm$ to $a(t=15)=0.4 mm$, where the ratio $R/a$ is $\sim0.7$. Our fit has a minimum at $t=15$ secs, which we take as the signal for the fly to stop extruding and start to ingest the droplet.

This yields excellent results for $H=0.6$, but fails for $H=0.8$. This is to be expected, since at this high humidity the fly actually discards the droplet, instead of ingesting it. Maintaining the same material parameters $z_{1},z_{2}$ and now fitting the drop parameters to the experimental data at $H=0.8$, shows that the fly starts with a $20\%$ smaller droplet. For some initial time $R$ is actually smaller than $a$, meaning that the fly has to distort the droplets spherical shape. In general, the congruence between our empirical results and those predicted by the model herein presented is illustrated at Fig. 2C (main text). Clearly, the proposed model was able to explain the empirical changes in temperature and, therefore, supports our proposition of an exponential activation of the evaporative cooling during the exhibition of the bubbling behaviour (Figure 3, main text).

Finally, we mention the neglect of a host of factors: labellum/proboscis contact and geometry, liquid/vapour boundary layer, the drop’s geometry etc. Although we expect these factors to influence the dynamic of evaporative cooling, our model is too crude to address them. Thus, more experimental information is required to refine the proposed model. However, the basic cooling effect produced by the crop fluid tidal movement will still be present, even if those factors are neglect.

Our computation used software Scilab 6.0.0, Free and Open Source software (distributed under CeCILL license - GPL compatible) developed by Scilab Enterprises.

**References**

1. N. A. Fuchs, Evaporation and droplet growth in gaseous media (Pergamon Press, Oxford, 1959).

2. H. R. Pruppacher, J. D. Klett, Microphysics of Clouds and Precipitation, (Springer science + Buisness Media B.V. 2010).

3. G. D Kinzer and R. Gunn, J. The evaporation, temperature and thermal relaxation-time of freely falling waterdrops. Meteorology, 8, 71 (1951).

**Figures**


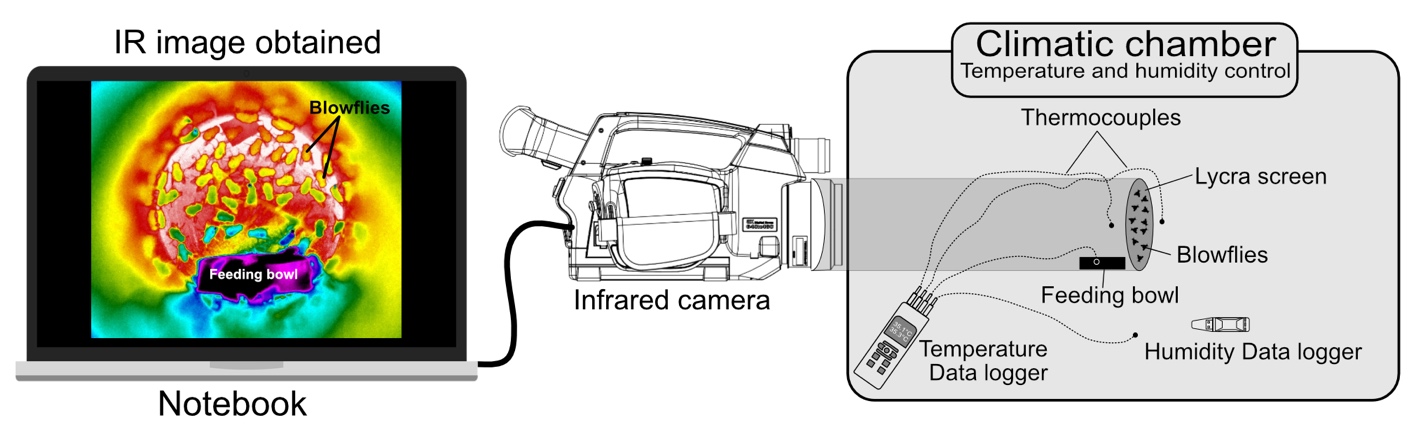


**Supplementary figure 1.** Infrared monitoring setup. Schematic representation of the experimental setup used to monitor changes in superficial body temperature of *Chrysomya megacephala* during the exhibition of the bubbling behaviour. This setup also allowed to test the influence of photoperiod, temperature, and relative humidity on the frequency and effectiveness of the behaviour.

**Video Legends**

**Video 1:**Infrared imaging video of the bubbling behaviour exhibited by *Chrysomya megacephala* (dorsal view).

Dorsal view of a group of flies (3 to 5 individuals) with 2 individuals performing the behaviour. At time 0004, one individual (middle right) starts extruding a droplet, in which it is joined by a second fly (middle left) at time 0013. The duet continues to exhibit the behaviour until approximately 0035, after which only the later persists until the end of the video. The impressive cooling of the droplet is immediately apparent from its bluish-darkening (see color temperature palette on the right) upon its extrusion, which spreads internally to the fly´s body, head-to-abdomen, as the droplet is moved inward. The bluish partial square on lower right is a food bowl, images on the left of the semicircular whitish line are reflections of the flies. Ambient temperature = 40°C and RH = 60%. Images acquired at 1 fps, video speed accelerated to 3.5Xs.

**Video 2:**Infrared imaging video of the bubbling behaviour exhibited by *Chrysomya megacephala* (lateral view).

Lateral view details of the temperature changes of the fluid droplet and fly´s body surface during five outward-inward movements of the droplet. Left image is a reflection, color temperature palette on the right, ambient temperature = 35°C and RH = 60%. Images acquired at 1 fps, video speed accelerated to 3.5Xs.

**Video 3:**X-ray computerized microtomography imaging of the cephalic region of *Chrysomya megacephala* during the exhibition of the bubbling behaviour.

Fly´s head is oriented to the left, leaving the anterior portion of the thorax visible to the right. Fluid droplet, compound eye, antennas, buccal apparatus, flight muscles, and brain are identified at the beginning of the video. The droplet is supported by the fly´s buccal parts, especially the labellum and expands-shrinks as fluid is moved outward-inward the oesophagus/foregut, whose expansion is visible as a darkening in the region just below the brain, corpora allata and cardiaca. Images acquired at 50kV, 200uA and 10W. Video acquired and displayed at 30 fps.

**Video 4:**Infrared imaging video of the bubbling behaviour exhibited by *Chrysomya megacephala* in response to photophase transition.

Change in the frequency of the behaviour in response to photophase transition. Video starts under lighted conditions and, as the lights are turned off at time 0020, the number of flies performing the behaviour increases. Ambient temperature = 30°C and RH = 60%. Images acquired at 3 fps, video speed accelerated to 720Xs.

**Video 5:**Infrared imaging video of the bubbling behaviour exhibited by *Chrysomya megacephala* (RH = 80%).

Disruption of the evaporative cooling effect of the behaviour due to high ambient relative humidity (RH = 80%). The behaviour is performed by three different flies (identified by arrows). Notice that the fluid tidal movements are not accompanied by the cooling of the droplet and of the fly´s body surface temperature (for comparison, see Video 1). In fact, after a few outward-inward movements, the fly spits the droplet out to the substrate. Color temperature palette on the right, ambient temperature = 35°C. Images acquired at 1 fps, video speed accelerated to 3.5 Xs.
